# The PFKFB3 Inhibitor AZ67 Inhibits Angiogenesis Independently of Glycolysis Inhibition

**DOI:** 10.3390/ijms22115970

**Published:** 2021-05-31

**Authors:** Besa Emini Veseli, Pieter Van Wielendaele, Mirela Delibegovic, Wim Martinet, Guido R. Y. De Meyer

**Affiliations:** 1 Laboratory of Physiopharmacology, University of Antwerp, 2610 Antwerp, Belgium; besa.emini@uantwerpen.be (B.E.V.); wim.martinet@uantwerpen.be (W.M.); 2 Aberdeen Cardiovascular and Diabetes Centre, Institute of Medical Sciences, University of Aberdeen, Aberdeen AB25 2ZD, UK; m.delibegovic@abdn.ac.uk; 3 Laboratory of Medical Biochemistry, University of Antwerp, 2610 Antwerp, Belgium; Pieter.VanWielendaele@uantwerpen.be

**Keywords:** AZ PFKFB3 67, PFKFB3, endothelial cells, angiogenesis

## Abstract

Angiogenesis is the process of new blood vessel formation. In this complex orchestrated growth, many factors are included. Lately, focus has shifted to endothelial cell metabolism, particularly to the PFKFB3 protein, a key regulatory enzyme of the glycolytic pathway. A variety of inhibitors of this important target have been studied, and a plethora of biological effects related to the process of angiogenesis have been reported. However, recent studies have disputed their mechanism of action, questioning whether all the effects are indeed due to PFKFB3 inhibition. Remarkably, the most well-studied inhibitor, 3PO, does not bind to PFKFB3, raising questions about this target. In our study, we aimed to elucidate the effects of PFKFB3 inhibition in angiogenesis by using the small molecule AZ67. We used isothermal titration calorimetry and confirmed binding to PFKFB3. In vitro, AZ67 did not decrease lactate production in endothelial cells (ECs), nor ATP levels, but exhibited good inhibitory efficacy in the tube-formation assay. Surprisingly, this was independent of EC migratory and proliferative abilities, as this was not diminished upon treatment. Strikingly however, even the lowest dose of AZ67 demonstrated significant inhibition of angiogenesis in vivo. To our knowledge, this is the first study to demonstrate that the process of angiogenesis can be disrupted by targeting PFKFB3 independently of glycolysis inhibition.

## 1. Introduction

Angiogenesis is the growth of new blood vessels from preexisting vasculature. It is a complex multifaceted process that is critical for many physiological and pathological conditions such as embryonic development, wound repair, inflammation, and tumor growth [1]. In this dynamic process, the proliferation and migration of ECs and the recruitment of pericytes play a central role, constructing a vascular tube that is oriented toward the site that requires oxygen and nutrient supply [2].

ECs compose the inner layer of the (cardio)vascular system and play an important role in maintaining oxygen and nutrient supply to all tissues in the body. Under physiological conditions, adult ECs remain largely in a quiescent state, but can become rapidly activated in response to pathological conditions. In these conditions, e.g., injury, where the formation of new blood vessels is required, the process of angiogenesis is activated. Three main EC subtypes govern the process of angiogenesis: migratory tip cells, which are specialized in growing vascular sprouts in response to growth factors; stalk cells, which proliferate and elongate the sprout; and quiescent phalanx cells, mostly present in established vessels and are responsible for maintaining vascular homeostasis and endothelial barrier function [3,4,5].

Different activators of angiogenesis have been described thus far, with the most well-characterized being vascular endothelial growth factor (VEGF). Two receptors mediate the effects of VEGF, namely VEGF receptor 1 and VEGF receptor 2 [6], with the latter having a higher sensitivity and responsiveness to VEGF. For several years, blocking VEGF and/or its respective receptors has been a strategy for targeting pathological angiogenesis, but drug resistance and insufficient efficacy have limited its success, suggesting that alternative anti-angiogenic strategies are required [7,8,9].

Recently, attention has shifted to EC metabolism. Our understanding of this topic has grown, and it has become clear that cellular metabolic pathways act as central mediators of signaling and angiogenesis. In general, contrary to other healthy cells [10,11], ECs, independently of their subtype, being arterial, venous, lymphatic, or microvascular ECs are highly glycolytic. Furthermore, the glycolysis rate in these cells is comparable to the rate measured in various tumor cells [11]. A crucial step in the glycolysis pathway is the phosphofructokinase reaction (PFK1) which converts fructose-6-phosphate (F6P) to fructose-1,6-bisphosphate (F-1,6-P_2_). Besides PFK1, phosphofructokinase-2/fructose-2,6-bisphosphatase (PFKFB) enzymes synthesize fructose-2,6-bisphosphate (F-2,6-P_2_), an allosteric activator of PFK-1 and the most potent stimulator of glycolysis [12]. The PFKFB family consist of four isoforms, named PFKFB1-PFKFB4, that are involved in both synthesis and degradation of F-2,6-P_2_ [13,14,15,16]. These bifunctional isoenzymes display distinct properties, including tissue expression profiles, the ratio of kinase/phosphatase activities, and their responses to protein kinases, hormonal and growth factor signals [17,18]. Thus, only PFKFB3 reveals a kinase to phosphatase ratio (K:P) of about 740:1, favoring the formation of F-2,6-P_2_ and therefore sustaining high glycolytic rates [19,20]. Albeit being a ubiquitous enzyme [21], under physiological conditions PFKFB3 has a variable low basal expression in all organs. It has been well documented that *pfkfb3* gene expression levels are upregulated in response to growth factors (VEGF, PDGF, FGF2, insulin) [22,23], proinflammatory cytokines (TNF-α, IL-β, TGF-β1) [24,25,26], hypoxia [27,28], or different stress stimuli [29]. However, under different stimuli, the mechanisms involved in PFKFB3 regulation in different cell types differ.

With accumulating knowledge of PFKFB3 in angiogenesis, there has been an increased interest in the identification and development of PFKFB3 inhibitors. 3PO, the most well-studied, and its derivatives PFK15 and PFK158, belong to this class. A range of biological activities have been attributed to these compounds, including reduction of F-2,6-P_2_ levels, inhibition of glucose uptake and lactate production, reduction in glycolytic flux, and induction of apoptosis in cancer cell lines both in vitro and in vivo [30,31]. Inhibition of EC proliferation and migration, resulting in reduced vessel sprouting in EC spheroids, zebrafish embryos, and the postnatal mouse retina, have all been attributed to 3PO [30,32]. However, questions were raised early on in the literature as to whether all these effects are attributable solely to PFKFB3 inhibition. Research groups have disputed that 3PO acts as a PFKFB3 inhibitor, as this compound is inactive in a PFKFB3 kinase assay, a lack of crystal structure could not confirm binding [33], and cytotoxicity could not be distinguished from glycolytic cellular activity for the concentrations used [34]. Our group has clearly reported that there is no binding between 3PO and the PFKFB3 protein [35]. PFK15 has shown a much higher IC_50_ towards the PFKFB3 enzyme compared to what has been reported (IC_50_ > 1000 µM versus 0.2 µM), while PFK158 has no effect on PFKFB3 enzymatic activity [36]. All in all, reported data on PFKFB3 inhibition rely on compounds with low specificity so that additional off targets could not be ruled out. Therefore, in this present study, we used the small molecule AZ67, a bona fide PFKFB3 inhibitor with high potency and selectivity for this target. By using isothermal titration calorimetry, we demonstrated binding of AZ67 to PFKFB3. Importantly, we showed that targeting PFKFB3 leads to angiogenesis inhibition in vitro and in vivo, independently of glycolysis inhibition. However, it was surprising that all these effects occurred with no impact on EC proliferation or migration abilities, thus providing novel insights into the angiogenesis inhibition process.

## 2. Results

### 2.1. AZ67 Binds to Human Recombinant PFKFB3 Enzyme

**Figure 1:**
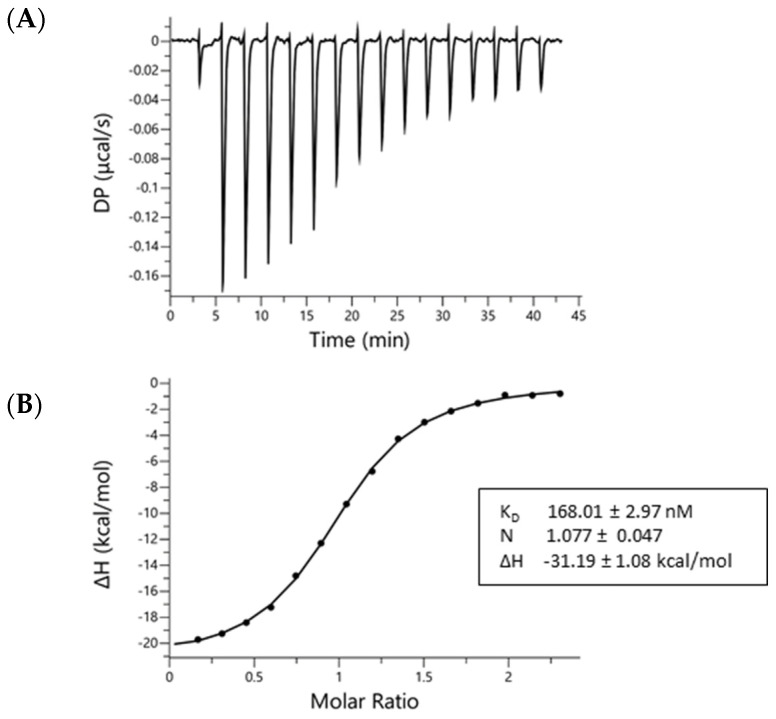
AZ67 binds to PFKFB3. Binding of AZ67 to PFKFB3 was analyzed via isothermal titration calorimetry using a MicroCal Peaq-ITC isothermal titration calorimeter. (**A**) Raw thermogram; (**B**) integrated injection heat (kcal/mol) as function of the molar ratio. The obtained fitted values in the box are the mean ± SEM of three replicates.

Considering the latest finding that 3PO is not a PFKFB3 inhibitor [35], we aimed to demonstrate that AZ67 binds to PFKFB3. By using isothermal titration calorimetry, we verified and characterized the binding between AZ67 and PFKFB3. Figure 1 demonstrates a raw thermogram (top panel), and integrated data, indicating clear binding and saturation towards the end of the assay run. Mean values (±SEM) for the KD (168.01 ± 2.97 nM), N-value (1.077 ± 0.047) and binding enthalpy (−31.19 ± 1.08 kcal/mol), reveal that the binding had a dissociation constant in the nanomolar range, with a 1:1 stoichiometry between AZ67 and the PFKFB3-monomers (thus 1 AZ67 molecule binding to 1 PFKFB3 monomer).

### 2.2. HAOECs Viability Was Not Affected by AZ67

**Figure 2:**
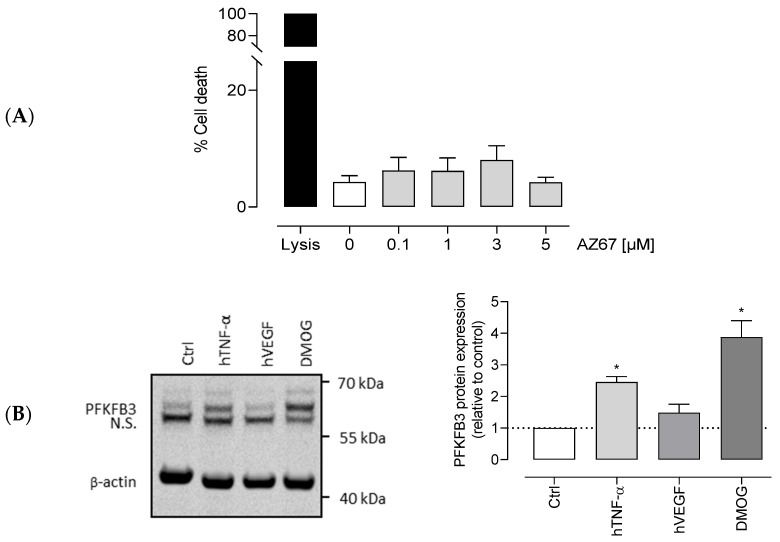
Viability of HAOEC upon AZ67 treatment and PFKFB3 expression with different stimuli. (**A**) AZ67 does not affect the viability of HAOEC. Cells were treated for 24 h with either vehicle or different concentrations of AZ67 (0.1, 1, 3, 5 µM). AZ67did not show cytotoxic effects up to 24 h treatment compared to vehicle. Data are represented as mean ± SEM, N = 5, *p* > 0.05 (one-way analysis of variance (ANOVA)). (**B**) PFKFB3 protein expression in HAOEC. Western blot analyses and densitometric quantification of PFKFB3 protein levels in HAOEC lysates with different stimuli. HAOECs were seeded in 24-well plates and further stimulated with 20 ng/mL hTNF-α, 10 ng/mL hVEGF, or 1 mM DMOG for 24 h. Expression was normalized per independent experiment to β-actin and expressed relative to control. All data points represent normalized averages obtained from 3–4 independent experiments and are presented as mean ± SEM. N.S—nonspecific band. * *p* < 0.05 vs. control (one-way ANOVA, followed by the Dunnett T3 test).

Before proceeding with other in vitro assays, we tested the possible toxicity of AZ67. As shown in (Figure 2A), AZ67 did not affect the viability of human aortic ECs (HAOECs) up to a concentration of 5 µM.

### 2.3. VEGF, TNF-α, and DMOG Upregulate Endothelial PFKFB3

Much is already known about PFKFB3 protein expression in cells, a ubiquitous enzyme with a variable low basal expression in all organs under physiological conditions. As mentioned in the introduction, an upregulation in response to pathological-mimicking conditions has already been documented. However, as under different stimuli the mechanisms involved in PFKFB3 expression in different cell types differ, it was important to demonstrate protein expression in HAOECs, the cells of choice for this study. To do so, we examined the effects of angiogenic factor VEGF, and of the pro-inflammatory cytokine TNF-α on PFKFB3 protein expression. As shown in (Figure 2B), after a 24 h stimulation, the expression of PFKFB3 in these cells was 2 to 3 times higher with 20 ng/mL hTNF-α stimulation, while 10 ng/mL hVEGF had no effect in this regard. In addition, DMOG, an oncometabolite mimetic known to significantly upregulate PFKFB3 protein expression (in other cell types) [37], showed an even higher induction of protein expression. When using 1 mM DMOG for 24 h, protein expression was upregulated up to 5 times. 

### 2.4. AZ67 Does Not Inhibit L-Lactate Production, Nor ATP Levels in Activated HAOECs

**Figure 3:**
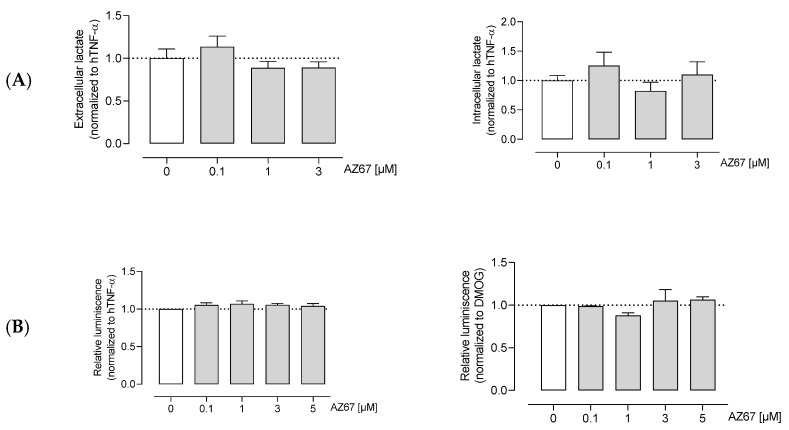
AZ67 does not inhibit glycolysis in ECs. (**A**) HAOEC were cultured and treated with AZ67 (0.1, 1, 3 µM) supplemented with 20 ng/mL hTNF-α for 16 h. Extracellular and intracellular L-lactate was measured to evaluate glycolysis. Data represented as mean ± SEM; N = 6, *p* > 0.05 (one-way ANOVA). (**B**) Total cellular ATP was measured using the CellTiter-Glo assay. Cells were stimulated with 20 ng/mL hTNF-α (**left**), or 1 mM DMOG (**right**) for 24 h, followed by treatment with AZ67 (0.1, 1, 3, 5 µM) with the concomitant addition of hTNF-α or DMOG for an additional 24 h. Data represented as mean ± SEM; N = 4–5, *p* > 0.05 (one-way ANOVA).

Next, we aimed to elucidate whether AZ67 inhibits glycolysis. Knowing that ECs are highly glycolytic and for ATP production rely on glycolysis rather than oxidative phosphorylation, measuring the end product of anaerobic glycolysis as well as ATP levels is useful for detecting shifts in glucose metabolism. To this end, AZ67 did not affect extra- and intracellular L-lactate levels in activated HAOECs (Figure 3A). The same was found for the ATP levels, where no significant decrease upon AZ67 treatment was noticeable (Figure 3B).

### 2.5. AZ67 Inhibits Endothelial Cell Tube Formation on TNF-α Activated Cells, but Does Not Inhibit Endothelial Cell Proliferation and Migration

**Figure 4:**
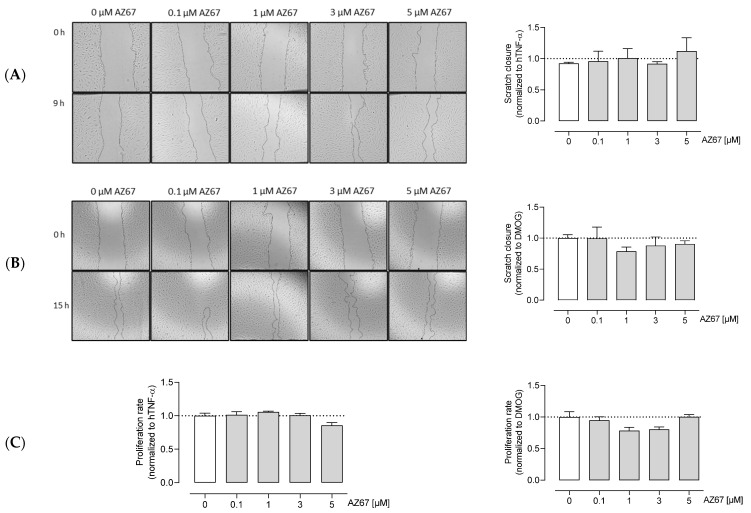
AZ67 does not inhibit the migration and proliferation of HAOEC. Cells were pre-treated with 20 ng/mL TNF-alpha (**A**), or 1 mM DMOG (**B**) for 24 h. Each well was marked below the plate surface by drawing a vertical line. A scratch intercepting the marked line was done in each well, cells were rinsed and medium containing different concentrations of AZ67 (0.1, 1, 3, 5 µM) together with 20 ng/mL hTNF-α, or 1 mM DMOG was added. The wound area was measured at 0 and 9 h (for TNF-α treated), and 15 h (for DMOG treated) and migration was quantified. N = 3, *p* > 0.05 (one-way ANOVA). (**C**) HAOEC were stimulated with 20 ng/mL hTNF-α (**left**), or 1 mM DMOG (**right**) and treated with AZ67 at different concentrations (0.1, 1, 3, 5 µM). After 24 h of incubation time, WST-1 reagent was added to all wells and incubated for an additional 4 h. WST-1 cleavage product was measured using a microplate reader. N = 4, *p* > 0.05 (one-way ANOVA).

**Figure 5:**
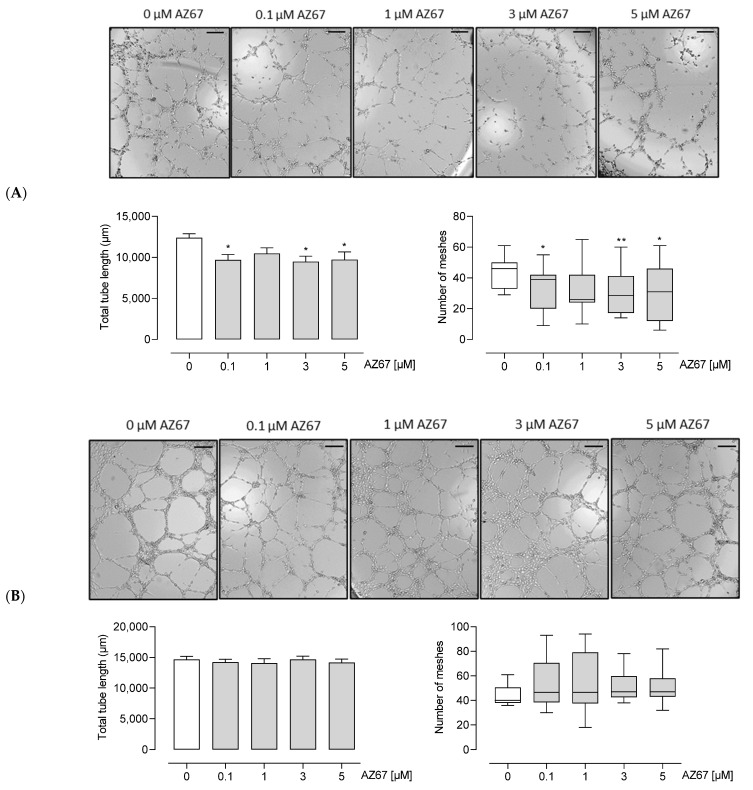
AZ67 inhibits tube and mesh formation of HAOEC upon TNF-α (**A**) but not DMOG (**B**) pre-treatment. ECs were pre-treated with 20 ng/mL hTNF-α, or 1 mM DMOG for 24 h. Cells were seeded onto a Matrigel matrix and treated with different concentrations of AZ67 (0.1, 1, 3, 5 µM) supplemented with hTNF-α, or DMOG for 8-9 h. Representative images of tube and mesh-like structures were captured using an inverted microscope. Scale bar = 200 µm. Tube length: * *p* < 0.05 vs. 0 µM (ANOVA, followed by Dunnett test); Number of meshes * *p* < 0.05, ** *p* < 0.01 vs. 0 µM, Kruskal–Wallis test, followed by Mann–Whitney test.

Next, we investigated whether AZ67 has any effects on HAOECs proliferation and migration, the two important steps in new vessel formation (the process of angiogenesis). As shown in (Figure 4A,C—left), AZ67 did not inhibit the process of migration, nor proliferation, in activated ECs. This effect was also not present in HAOECs pre-treated with the oncometabolite mimetic DMOG (Figure 4B,C—right). To further explore whether PFKFB3 inhibition affects neoangiogenesis, an in vitro Matrigel assay was performed. This assay is based on the ability of HAOECs to form tube and mesh structures when seeded on a growth factor-enriched matrix. As PFKFB3 protein expression was highest upon TNF-α and DMOG stimulation/treatment, these two conditions were explored further for this experiment. Interestingly, upon TNF-α stimulation and AZ67 treatment, a significant reduction in tube and mesh structure formation was observed (Figure 5A). However, when cells were pre-treated with 1 mM DMOG, AZ67 did not inhibit this process and a healthy network of chord structures was formed independently of the AZ67 concentration used (Figure 5B).

### 2.6. In Vivo Angiogenesis Is Inhibited upon AZ67 Treatment

**Figure 6:**
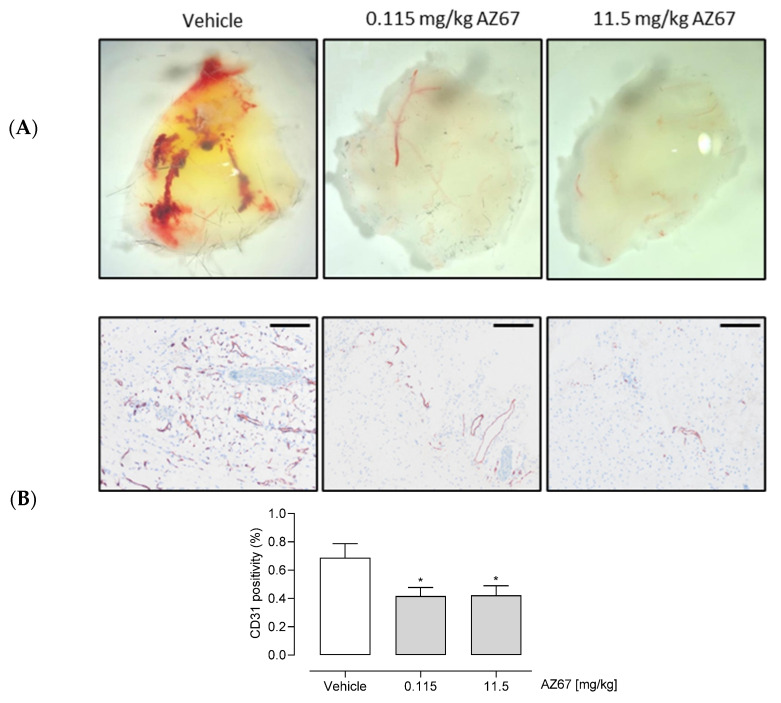
AZ67 inhibits (neo)angiogenesis in vivo. (**A**) Representative macroscopic images of the plugs from the in vivo Matrigel assay. Growth factor reduced Matrigel was mixed with either vehicle, or an equal amount of volume of AZ67 in two different doses: 0.115 mg/kg body weight, and 11.5 mg/kg body weight. The mixture was injected subcutaneously into the flank area of 6-week-old C57BL/6 mice, while under anesthesia. Two weeks after injection the mice were sacrificed, and plugs were harvested for immunohistochemistry processing. (**B**) Top panel: representative macroscopic images of the Matrigel sections. Anti-CD31 antibody was used to delineate ECs. Images were acquired using an Olympus BX43 Microscope. Bottom panel: quantification of CD31 positivity of the microscopic images. Scale bar = 200 µm. * *p* < 0.05 vs. vehicle (one-way ANOVA, followed by Dunnett test).

Finally, we aimed to elucidate whether pharmacological inhibition of PFKFB3 by AZ67 would result in the expected in vivo effects for neovessel formation. Considered as the standard method for in vivo evaluation of pro- and anti-angiogenic compounds, the Matrigel plug assay revealed that AZ67 significantly inhibited new vessel formation in C57BL/6 mice. Fourteen days after implantation, the Matrigel plug containing the vehicle formed a large blood vessel network connected with the host vasculature, with the vessel containing blood, as seen in (Figure 6A) (red structures). In contrast, the Matrigel plug containing AZ67 displayed no functional vessels. This was further represented by microscopic images of CD31 positive vessel structures (in brown), accompanied by the quantification (Figure 6B).

## 3. Discussion

We evaluated AZ67 activity on the angiogenesis process. AZ67, which is a small molecule inhibitor with high potency and selectivity for isoform 3 of the PFKFB family, showed a remarkable ability to alter angiogenesis in vitro as well as in vivo. Importantly, all this occurred by using AZ67 at concentrations as low as 100 nM in in vitro assays (approximating the determined KD value from Figure 1), and 115 µg/kg body weight in an in vivo animal model. Surprisingly, the disrupted angiogenesis process resulted with no detectable inhibition of two preceding steps of angiogenesis, being proliferation and migration (of ECs). Interestingly, we have highlighted a novel approach for angiogenesis inhibition through the PFKFB3 target, with no detectable effects on the glycolytic pathway.

First, by using isothermal titration calorimetry, we demonstrated binding of AZ67 to the human recombinant PFKFB3 enzyme. Thereafter, stimulation with a pro-inflammatory cytokine, or treatment with the oncometabolite mimetic DMOG, demonstrated an evident upregulation of the PFKFB3 protein in HAOECs. Furthermore, in accordance with these results, we also found a significant decrease in tube- and mesh-like structure formation upon AZ67 in activated ECs. The process of angiogenesis encompasses a multitude of factors. ECs, as the harbingers of this chain, together with pericytes, play a central role [1,2]. During the process of angiogenesis, the dynamics between endothelial migratory (tip) and proliferatory (stalk) cells are cooperatively controlled by VEGF-A stimulation and DLL4-Notch signaling [38]. VEGF-A induces angiogenesis by stimulating EC migration and proliferation, while DLL4-mediated activation of Notch signaling in neighboring cells suppresses VEGFR2 expression and promotes a stalk cell phenotype [39]. Interestingly, even though Notch signals promote proliferation of ECs in vivo, the same effect leads to a reduction in EC proliferation in vitro [40]. In addition, as there is a relationship between this signaling pathway and PFKFB3-driven glycolysis [11], we can hypothesize that AZ67, through PFKFB3 (inhibition), could play a role in the upstream or downstream pathway of Notch signaling. ECs have the remarkable ability to switch metabolism according to their needs. Upon anaerobic glycolysis inhibition, ECs can shift towards side pathways such as the pentose phosphate pathway (PPP) and the hexosamine biosynthesis pathway (HBP) [41], which are very important for generating intermediates for cell growth and proliferation [42]. Upon PFKFB3 blockage by AZ67, a shift could have taken place, leading to uninterrupted proliferation and migration of ECs.

In their efforts to design potent and selective inhibitors of metabolic kinase PFKFB3, Boyd et al., reported a series of inhibitors with high selectivity over the related PFKFB3 isoform. Among these, AZ67 was the focus of our study, as it has a high selectivity for PFKFB3 compared to other isoforms, it decreases F-2,6-P_2_ levels, and it inhibits PFKFB3 kinase activity—showing good activity in the cell assays, albeit with no apparent effects on lactate production [33,36]. This latter effect, later also confirmed in astrocytes [36], was an interesting point when confirming angiogenesis reduction upon PFKFB3 blockage with no impact on the glycolysis pathway. This was important as other groups have reported that PFKFB3-driven glycolysis directly modulates vessel sprouting [11]. This shift could be attributed to the fact that other research groups used non-specific PFKFB3 inhibitors, where off-target effects could not be ruled out (3PO does not bind to PFKFB3; PFK158 has no effect on PFKFB3 enzymatic activity; PFK15 showed a much higher IC_50_ compared to what has been reported) [33,34,35,36].

Previous anti-angiogenic therapies have attempted to inhibit vascular growth signals, such as VEGF, but their success was limited due to cell adaptation and therapy-resistance [7,8,9]. The shift to EC metabolism seems promising, especially after demonstrating that PFKFB3 is the driving force, driving vessel sprouting in response to genetic signals [11]. The most well-studied compound in this regard, 3PO, displays a 30–40% reduction in glycolysis in treated ECs in vitro, resulting in decreased proliferation, migration and sprouting. However, as the latest findings revealed that PFKFB3 is not the primary target, if any, of 3PO, with a much higher attribution to monocarboxylate transporter blockage [35], additional avenues need to be explored.

In vivo, AZ67 showed a remarkable inhibitory potency by reducing angiogenesis formation with a dose as low as 115 µg/kg of body weight. Compared to other anti-angiogenic therapies which require a range of mg/kg dosing regimens [43], this provides new hope for designing treatments for pathological angiogenesis, with very low to limited adverse effects.

Despite the variety of angiogenic mechanisms, most of the current research is focused on the mechanism of sprouting angiogenesis because this mechanism was first described and because most existing experimental models are related to sprouting angiogenesis [44]. Consequently, the mechanism of intussusceptive angiogenesis is often overlooked in angiogenesis. However, in intussusceptive angiogenesis, also known as splitting angiogenesis, a new blood vessel is created by splitting of an existing blood vessel into two [45,46,47]. Intussusceptive angiogenesis is currently considered an important alternative and complementary form of sprouting angiogenesis [48]. Because it remains elusive whether this type of angiogenesis is affected by AZ67, the present in vitro and in vivo observations could be complemented in future studies using other markers and electron microscopy that make it possible to follow the morphofunctional basis of the different types of angiogenesis as well as the behavior of the other components of the vessel wall, including pericytes [49]. Indeed, Folkman and Haudenschild dispute on the necessity of pericytes in capillary tube formation in vitro [50]. These cells play an important role in in vivo chain [2], thus an interplay of these components in an appropriate animal model, be it cancer-based or other excessive neovascularization related disease-based, remains an attractive aspect for future studies.

Taken together, our findings provide novel insights into a particular approach for targeting angiogenesis-related diseases, such as cancer, and this could also be extended to other diseases with excess neovascularization. Even though PFKFB3-inhibiton has been demonstrated in anti-angiogenic approaches, its association with glycolysis inhibition has been intertwined. Here, a glycolysis-independent angiogenesis inhibition was demonstrated. Most importantly, the inhibitor used in this study exceeds the already known angiogenesis inhibitors in terms of dose-efficacy (regimen), while the level of angiogenesis inhibition does not surpass 20–30%, alluding to even lower expected side effects.

## 4. Materials and Methods

### 4.1. Isothermal Titration Calorimetry (ITC)—Binding Assay

Binding of AZ PFKFB3 67 (abbreviated as AZ67, Tocris, Bio-Techne Ltd, Abingdon, UK) to PFKFB3 was analyzed by isothermal titration calorimetry using a MicroCal Peaq-ITC isothermal titration calorimeter (non-automated version, Malvern Pananalytical Ltd, Malvern, UK). Prior to ITC analysis, recombinant human PFKFB3 produced in *E. coli* (Flemish Institute for Biotechnology (VIB), Protein Service Facility, University of Ghent, Ghent, Belgium) was dialyzed against 2 L buffer (20 mM Tris, 500 mM NaCl, 5 mM MgCl_2_, 2 mM DTT at pH 7.4) for 2 h at 4 °C under constant stirring, followed by switching to a novel 2 L buffer vial and overnight dialysis. For this purpose, Slide-A-Lyzer Dialysis G2 cassettes (Thermo Scientific, Waltham, MA, USA) were used. The concentration of the sample after dialysis was determined by UV-absorbance at 280 nm using a Spectramax Plus 384 (Molecular Devices, San Jose, CA, USA). A small amount of the second dialysis buffer volume was kept for matching the ligand solutions (AZ67). All buffers were prepared in ultrapure water (18.2 MΩ.cm), equilibrated to room temperature and degassed for 15 min in an ultrasonication bath before use.

The reference cell of the Peaq-ITC was filled with degassed ultrapure water. 40 µM of AZ67 was titrated into 3.4 µM recombinant PFKFB3. Due to the solubility properties of AZ67 and the need to match titration solutions, both solutions contained 1.01% DMSO and 0.05% Ethanol. The PFKFB3 solution was put in the sample cell after 2 min of pre-equilibration with the assay buffer (same recipe as dialysis buffer). The ligand solution was administered in the injection syringe. Titration conditions were as follows: one initial injection of 0.4 µL was followed by 15 injections of 2.5 µL. The initial spacing was set to 180 s, while the remaining spacing was set to 150 s. The sample cell was continuously stirred at 750 rpm. Temperature was set at 37 °C before loading and kept constant during the complete assay run. The DP (differential power between the reference and sample cells to maintain a zero-temperature difference between the cells) was set to 5.

To determine the dilution heats, a control titration was performed consisting of injections of ligand into the buffer-filled cell (thus in the absence of PFKFB3, without binding). Thermograms were analyzed using the Microcal Peaq-ITC Analysis software, using the ‘one set of sites’ binding model, by including the corresponding control titration. The AZ67-PFKFB3 titration was run in triplicate. Fitting results were verified by repeating data analysis using Nitpic-Sedphat and Origin 7, confirming the accuracy of the MicroCal Analysis software fitting (data not shown).

### 4.2. Western Blot Analyses

Human aortic ECs (HAOECs) were purchased from Sigma Aldrich (304–05a) and were used at passages 3 to 9. HAOECs were cultured in Endothelial Cell growth Medium (Sigma-Aldrich, St. Louis, MO, USA) supplemented with 1% penicillin/streptomycin. To investigate the expression of PFKFB3 protein, HAOECs were seeded into 24-well plates in complete medium until 80–90% confluency. Cells were stimulated with different stimuli (20 ng/mL hTNF-α (PHC3015; Fisher Scientific), or 10 ng/mL hVEGF (PHC9394; Fisher Scientific, Waltham, MA, USA), or 1 mM dimethyloxalylglycine—DMOG (71210-50; Sanbio, Cayman Chemical, Ann Arbor, MI, USA)) for 24 h. After washing with ice-cold PBS, cells were lysed in an appropriate volume of Laemmli sample buffer containing β-mercaptoethanol and boiled for 5 min. Protein samples were then loaded on precast Bolt 4–12% Tris-Bis gels and after electrophoresis transferred to Immobilon-FL PVDF membranes using pre-mixed Tris/glycine transfer buffer. The membranes were blocked for 1 h with Odyssey blocking buffer diluted in PBS (1:5). An overnight incubation at 4 °C with primary antibodies (anti–β-actin (ab8226; Abcam, Cambridge, UK), and anti-PFKFB3 antibody (ab181861; Abcam, Cambridge, UK)), was followed by a 1 h incubation with IRDye-labeled antibodies (goat anti-mouse IgG, 926-68070, and goat anti-rabbit IgG, 926-32211) at room temperature. Antibody detection was achieved using an Odyssey SA infrared imaging system (LI-COR Biosciences, Lincoln, NE, USA) with Image Studio Software used as a tool to quantify the intensity of the protein bands.

### 4.3. Viability Assay

Cell viability was measured using a ToxiLight bioassay Kit (Lonza, Basel, Switzerland), following the manufacturer’s protocol. Briefly, HAOECs resuspended in Endothelial Cell Growth Medium, supplemented with 1% antibiotics were plated into 96-well plates (5 × 10^4^ cells/mL), and subsequently treated with vehicle or different concentrations of AZ67 (0.1, 1, 3, 5 µM). After 24 h, 20 µL of cell supernatant was transferred to a luminescence compatible 96-well plate. One hundred microliters of AKDR reagent was added, following an incubation of 5 min and luminescence was measured using a Luminoskan plate reader on a 1 s integrated time reading per sample.

### 4.4. L-Lactate Measurements

Intracellular and extracellular lactate levels were measured in vitro using an L-lactate Assay Kit (Cayman Chemical, Ann Arbor, MI, USA) according to the manufacturer’s protocol. HAOECs were seeded into a 12-well plate in Endothelial Cell Growth Medium supplemented with 1% antibiotics, and upon 80–90% confluency cells were treated with different concentrations of AZ67 (0.1, 1, 3 µM) supplemented with 20 ng/mL hTNF-α. After a 16 h incubation time, the supernatant was removed to quantify extracellular L-lactate levels, while the cell pellet was used for intracellular L-lactate determination. A cell count was performed with an automated cell counter (Countess^®^ II FL, Life Technologies, Carlsbad, CA, USA). After deproteinization with cold metaphosphoric acid (0.25 M for cell pellet, and 0.5 M for supernatant), potassium carbonate (5 M) was added to neutralize the acid. Following centrifugation (10,000× *g* for 5 min) at 4 °C, the supernatant was assayed. Lactate fluorescent substrate was used as a fluorophore while fluorescence (λex = 540 nm; λem = 595 nm) was measured and normalized to the cell number.

### 4.5. ATP Assay (Measurements)

Analysis of total cellular ATP was performed using the CellTiter-Glo^®^ assay, according to the manufacturer’s protocol. HAOECs seeded at a cell density of 5 × 10^4^ cells/mL, were stimulated with 20 ng/mL hTNF-α, or 1 mM DMOG for 24 h. Cells were then treated with different concentrations of AZ67 (0.1, 1, 3, 5 µM) supplemented with either hTNF-α, or DMOG. After 24 h of incubation, cells were lysed using the CellTiterGlo reagent, while intensively shaken for 2 min. Cell lysates were transferred into the wells of white 96-well plates (PerkinElmer, Waltham, MA, USA) and read on a Luminoskan plate reader under standard luminescence settings.

### 4.6. Proliferation and Migration Assays

- Proliferation assay

Proliferation activity of HAOECs with AZ67 treatment was monitored using a colorimetric WST-1 assay according to the manufacturer’s instructions (Sigma-Aldrich, St. Louis, MO, USA). HAOECs were seeded into a 96-well plate, and upon reaching 80–90% confluency, stimulated with 20 ng/mL hTNF-α, or 1 mM DMOG, and AZ67 was added at the stated concentrations. After a 24 h incubation time, WST-1 reagent was added to all wells at a 1/10 dilution and incubated for an additional 4 h. WST-1 cleavage product was measured at 450 nm (sample) and at 650 nm (background) using a microplate reader. WST-1 plus medium alone served as blank, which was subtracted from all values. The proliferation rate was calculated using the following formula: (WST-1 value/diluent control) × 100.

- Scratch assay

The wound healing assay, also known as the scratch assay, is a standard in vitro technique for determining collective cell migration [51]. For this assay, HAOECs plated at 5 × 10^4^ cells/mL were grown to confluency in Endothelial Cell Growth Medium supplemented with antibiotics. Subsequently, cells were stimulated with 20 ng/mL hTNF-α, or 1 mM DMOG for 24 h. Each well was marked below the plate surface by drawing a vertical line. A scratch intercepting the marked line was done in each well using a 200 µL sterile tip. Cells were rinsed with PBS to remove cell debris, and medium containing indicated concentrations of AZ67 together with hTNF-α, or DMOG was added. Photos of scratches were taken at timepoint 0 h, and after 9 h (hTNF-α treated) or 15 h (DMOG treated) incubation. The migration rate was calculated using the following formula: % closure = ((scratched area at 0 h—scratched area at 9 or 15 h)/scratched area at 0 h) ×100.

### 4.7. In Vitro Angiogenesis Assay

To visually analyze the anti-angiogenic ability of AZ67, an in vitro tube formation assay was performed. Briefly, 10 µL of Growth Factor Reduced Matrigel matrix (Corning^®^, Corning, NY, USA) was transferred to ibidi angiogenesis µ-slides (Ibidi GmbH, Grafelfing, Germany) and then incubated at 37 °C for 1 h. The HAOECs (1 × 10^4^ cells/well) pre-treated with 20 ng/mL hTNF-α, or 1 mM DMOG for 24 h, were seeded onto Matrigel matrix with 50 µL DMEM supplemented with 10% FBS, and different concentrations of AZ67 respectively, with supplemental addition of 20 ng/mL hTNF-α, or 1 mM DMOG and incubated at 37 °C for 8–9 h. The tube- and mesh-like structures were photographed under an inverted microscope (Celena^®^ Digital Imaging System, Logos Biosystem, Villeneuve d’Ascq, France) at ×4 magnification, and the total tube length and number of formed meshes were analyzed using the angiogenesis analyzer of Image J.

### 4.8. In Vivo Angiogenesis (Matrigel Plug) Assay

The subcutaneous Matrigel plug assay in mice is the standard method for in vivo evaluation of the pro- and anti-angiogenic potential of different compounds. The assay was performed as described previously [52,53]. Briefly, Growth Factor Reduced Matrigel (Corning^®^, Corning, NY, USA), thawed overnight at 4 °C, was mixed with either vehicle (control group), or with an equal amount of volume of AZ67 compound (0.115 mg/kg body weight, or 11.5 mg/kg body weight). Six hundred microliters of this mixture was injected subcutaneously into the flank area of 6-week-old C57BL/6 mice (Charles River, Wilmington, MA, USA), while under anesthesia. Two weeks after injection, mice were sacrificed, Matrigel plugs were removed and photographed using a dissection microscope to show the extent of vascularization. Next, Matrigel plugs underwent immunohistochemistry processing (fixation in 2% paraformaldehyde for 24 h, followed by paraffin embedding, sectioning and staining). Anti-CD31 antibody (77699S; Cell Signaling, Beverly, MA, USA) was used to delineate ECs. Sections were examined by light microscopy and photos were taken using an Olympus BX43 microscope (×5 magnification). CD31 positivity was quantified using the ImageJ Macro language. All animal procedures were approved by the ethical Committee of the University of Antwerp (protocol code 2017-81 and date of approval: November 2017).

### 4.9. Statistical Analyses

All data are expressed as mean ± SEM. Statistical analyses were performed using SPSS software (version 25). Statistical tests are specified in the figure legends. Probability values <0.05 were considered to be significant.

## Data Availability

The data presented in this study are available on request from the corresponding author.
